# What neighbourhood factors support more transport walking as adults age? A five-wave longitudinal multilevel cohort study in Brisbane, Australia

**DOI:** 10.1136/bmjph-2025-003333

**Published:** 2026-09-04

**Authors:** Tharindu Niwarthana Bandara, Gavin Turrell, Alysha De Livera, Belen Zapata-Diomedi, Lucy Gunn

**Affiliations:** 1 RMIT University, Melbourne, Victoria, Australia; 2 La Trobe University, Melbourne, Victoria, Australia

**Keywords:** Epidemiology, Cardiovascular Diseases, Age Factors, Causality, Public Health

## Abstract

**Introduction:**

Transport walking is a simple, cost-effective and sustainable way to promote physical activity that integrates movement into daily routines. A supportive built environment, including walkable areas—consisting of high residential density, good street connectivity and mixed land-uses—is associated with greater transport walking, making it a critical area for urban planning and policy interventions. However, most studies on age differences in built environment and transport walking are cross-sectional, limiting causal inference and emphasising the need for longitudinal research to strengthen evidence for healthy ageing policies.

**Methods:**

This study longitudinally examines age interaction effects with built environment attributes on transport walking, incorporating time-varying exposures and confounders, and accounting for neighbourhood self-selection. Data were from the How Areas in Brisbane Influence HealTh and AcTivity study, a five-wave multilevel cohort study from (2007–2016). Analysis used participants residing at the same address throughout the study. Transport walking was self-reported and classified as walkers or non-walkers. Generalised linear mixed-effects models were used to assess associations between the built environment and transport walking, and their interaction with age, adjusting for sociodemographic factors, length of residence, neighbourhood preference and neighbourhood disadvantage.

**Results:**

Higher residential density, street connectivity, land-use mix and walkability were longitudinally associated with greater transport walking. Interaction effects showed that older adults were more likely than younger adults to engage in transport walking in denser, more walkable neighbourhoods.

**Conclusion:**

More walkable neighbourhood environments, characterised by higher residential density, street connectivity and land-use mix, were associated with a greater likelihood of transport walking over time. Moreover, the associations with density and walkability were stronger at older ages, suggesting that these attributes may play an important role in supporting healthy ageing.

**WHAT IS ALREADY KNOWN ON THIS TOPIC**

- Walkable environments with high residential density, good street connectivity and land-use mix are known to support transport walking. However, most studies are cross-sectional, limiting causal inference.
- Additionally, longitudinal evidence on age-specific effects among adults remains limited.

**WHAT THIS STUDY ADDS**

- This study provides longitudinal evidence that walkability and its components are associated with increased transport walking among mid- to older-aged adults.
- It shows that older adults benefit more from dense and walkable neighbourhoods than their younger counterparts.

**HOW THIS STUDY MIGHT AFFECT RESEARCH, PRACTICE OR POLICY**

- The findings strengthen the evidence base for urban design policies that promote walkable environments, particularly to support transport walking and healthy ageing among older adults.
- This highlights the value of age-sensitive planning to improve physical activity and population health.

## Introduction

 Physical inactivity is a major risk factor for cardiovascular disease, type 2 diabetes and certain types of cancer.^1–3^ As people age, they are less likely to meet recommended levels of physical activity,^4^ increasing the risk of chronic diseases, which in turn places a significant burden on healthcare systems and increases associated economic costs.^3,5^ Transport walking is an easy-to-access, cost-free and relatively safe physical activity compared with some other forms of exercise, making it a sustainable option that can be readily incorporated into everyday routines.^6^ For older adults who need to limit or stop driving due to vision problems, cognitive decline or lack of confidence, transport walking may be an appropriate mode of physical activity,^3^ though these conditions can also pose challenges for walking depending on their severity and the availability of supportive infrastructure. Therefore, recognising the factors that influence transport walking is crucial for creating effective interventions and policies aimed at encouraging active living and promoting healthy ageing.

Research consistently shows that built environment attributes—such as residential density, street connectivity^7^ and land-use mix—are positively associated with transport walking.^8–10^ Walkability, a composite measure incorporating these attributes, has also been linked to higher levels of transport walking.^11,12^

Further, the relationship between the built environment and transport walking may vary across adult age groups, with most evidence in the literature being cross-sectional. For example, Ghani *et al*^13^ found that the association between age and transport walking varied significantly across neighbourhoods: in some areas the age differences were large, in others small and in some neighbourhoods no association existed. Some evidence indicates that higher land-use mix may help reduce age-related disparities in walking levels.^14,15^ Higher walkability has been found to be positively correlated with increased transport walking across all ages; however, the association is notably stronger in middle-aged individuals (36–49 years) compared with younger (18–35 years) and older adults (50–65 years).^12^ Nevertheless, the longitudinal evidence on how these built environment attributes differently influence transport walking among different adult age groups is limited,^16^ which restricts causal inference to inform policies aimed at promoting healthy ageing.^17^

Most existing longitudinal studies on walking have overlooked neighbourhood self-selection effects,^16^ which can bias the relationship between neighbourhood attributes and walking. Neighbourhood self-selection refers to the tendency of individuals who prefer walking to choose neighbourhoods that are conducive to walking.^18^ Moreover, many existing longitudinal studies have not used time-varying exposure variables measured simultaneously with the outcome variable.^16^ Instead, findings are often based on exposures measured at a single time point, usually at the baseline. Using time-varying exposures not only enhances the reliability of the measurements, but also allows for temporal assessment of associations.^19^

In addressing these gaps in the literature, this study examines longitudinal associations between residential density, street connectivity, land use mix and walkability on transport walking, and investigates if these associations differ by age group among middle- to older-aged adults. It is hypothesised that the built environment attributes are associated differently with transport walking behaviour of adults in different age groups. The study uses data collected at five time points over 9 years, enabling stronger temporal inferences than cross-sectional associations. Furthermore, the analysis accounts for neighbourhood self-selection using a proxy measure of participants’ preferences for living in their neighbourhoods, reducing potential bias arising from this factor.

## Methods

### Study population and data source

Data for this study were obtained from the HABITAT project (How Areas in Brisbane Influence HealTh and AcTivity),^20^ a multilevel longitudinal investigation focused on adults aged 40−65 at baseline in 2007. The primary aim of HABITAT is to explore how various individual and contextual factors—including social, environmental, demographic and psychological influences—affect physical activity and sedentary behaviours over time. The study cohort was drawn to broadly reflect the demographic profile of the Brisbane population of the sampled age group at the baseline.^21^

### Sampling methodology

Details of the sampling design for the HABITAT study are available in previously published work.^22^ In summary, a stratified random sampling approach was used to select participants from Census Collection Districts (CCDs) across Brisbane. All 1625 CCDs were scored using the Australian Bureau of Statistics’ Index of Relative Socioeconomic Disadvantage^23^ and categorised into 10 deciles. From each decile, 20 CCDs were randomly selected, yielding a total of 200 neighbourhoods. Data were collected by distributing a structured self-administered questionnaire, developed by Dillman,^24^ via mail. The HABITAT questionnaire items used to construct the variables analysed in this study are presented in online supplemental table S1. The initial wave in 2007 achieved a 68.4% response rate, with 11 035 completed surveys from eligible individuals. Subsequent waves took place in 2009 (n=7866, 72.6%), 2011 (n=6900, 67.6%), 2013 (n=6520, 67.6%) and 2016 (n=5187, 58.8%).^20^

### Variables

#### Outcome

At baseline and during each follow-up wave, participants were asked to report the number of minutes they spent walking for transport in the last week, using the question: ‘What do you estimate was the total time that you spent walking for transport in the LAST WEEK?’. They were instructed to include only walking for purposes such as commuting or running essential errands (such as going to work, shops, groceries, banks), excluding any recreational walking. Moreover, previous studies observed that this transport walking measure is associated with multiple built environment attributes and sociodemographic factors, suggesting that it appropriately captures transport walking behaviour.^21,25,26^ Due to the highly skewed and zero-inflated nature of the data, the variable was converted into a binary outcome (walker vs non-walker) to facilitate analysis and interpretation. At each time point, participants who reported any amount of walking were classified as walkers, while those reporting zero minutes were considered non-walkers.

#### Exposures

##### Built environment

Residential density, street connectivity and land-use mix data were derived within a 1 km road network buffer surrounding each participant’s home address. Using a buffer that follows road networks is suitable for studies on transport walking, as it accounts for walkable roads and barriers like rivers or lakes.^27^ A 1 km distance was chosen as older adults may choose to walk if their destination is within this distance.^28^ These data were collected in coordination with the individual-level survey periods to maintain temporal alignment between neighbourhood characteristics and participant responses. These neighbourhood attributes were calculated using Geographic Information System software and Brisbane City Council data, as detailed in Turrell *et al*.^20^

Residential density and street connectivity were calculated within the buffer as the number of dwellings per hectare and the number of four-way intersections, respectively. Land-use mix was measured using an entropy index that reflected the distribution of land across various uses, including retail, office/business, leisure/recreation, residential and health/community services.^26^ A walkability index was constructed in accordance with the approach used in many other studies by combining standardised measures of residential density, street connectivity and land-use mix.^11,29,30^ Each component was standardised within survey waves by using within wave mean and SD and then averaged to form the walkability index, which was subsequently rescaled to range from 0 to 100 for ease of interpretation, with higher values indicating more walkable neighbourhoods.

##### Age

Age at baseline was derived from participants’ reported year of birth and treated as a continuous variable in years.

### Covariates

#### Sex

Participants indicated their sex by answering the question, ‘Are you male or female?’

#### Education

Baseline highest educational qualification was classified into four categories: bachelor’s degree or higher, diploma, certificate or high school only.^8,26^

#### Occupation

Participants’ occupations were classified into five groups: professional, white-collar, blue-collar, home duties and retired or not easily classifiable.^8,26^

#### Household income

Participants were asked to estimate their total household income before tax by selecting from 14 income brackets. For analytical purposes, these responses were consolidated into six categories: $A130 000 or more per year; $A129 999−$A72 800; $A72 799−$A52 000; $A51 999−$A26 000; $A25 999−$A0; and missing (which included those who did not respond, selected ‘Don’t know’ or chose ‘Don’t want to answer’).^8,26^

#### Length of residence

The number of years participants had lived at their address was recorded at baseline and treated as a continuous measure in all models.

#### Neighbourhood self-selection

Factor analysis with orthogonal varimax rotation was used to assess participants’ preferences for residing in walkable neighbourhoods. The analysis drew on baseline responses to a set of items scored on a 5-point Likert scale, ranging from 1 (not at all important) to 5 (very important), which asked participants: ‘How important were each of the following in your decision to move to your current suburb?’. The list of items included factors such as housing affordability, potential for investment, proximity to work, safety from crime, proximity to schools, ease of walking to destinations, access to childcare, nearness to the city, proximity to green spaces or bushland, proximity to public transport, closeness to open areas like parks, desire to live near shops, accessibility to freeways/main roads, proximity to recreational facilities, moving in with a partner/spouse, sense of community and closeness to family members.

Four factors were identified with eigenvalues greater than 1, accounting for 43% of the total variance in responses for the items. The factor that accounted for the largest share of variance (14%) was linked to preferences for living in walkable areas—such as proximity to public transport, desire to live near shops and ease of walking to destinations. This factor demonstrated strong internal consistency, with a Cronbach’s alpha of 0.81.^31^ A latent variable representing neighbourhood preference was created from the factor scores and rescaled to range from 0 to 100, with higher scores indicating a stronger preference for attributes that promote transport walking. This measure was used as a continuous covariate in the regression models.^8^

#### Neighbourhood disadvantage

A socioeconomic score was assigned to each of the 200 neighbourhoods using the Index of Relative Socioeconomic Disadvantage (IRSD) developed by the Australian Bureau of Statistics.^32^ This index represents the overall level of disadvantage in an area, considering various socioeconomic factors, including education, occupation, income, unemployment and household tenure. The scores range from 1, indicating the most disadvantaged area, to 100, representing the least disadvantaged.^33^

### Statistical analysis

Statistical analysis was conducted using R software.^34^ The analytical sample excluded individuals who changed their residential address within the study period, as relocation could be driven by unobserved factors linked to both neighbourhood choice and walking behaviour.^35,36^ Additionally, participants were excluded if a different household member responded on their behalf in any wave, if they did not provide transport walking data for all waves, or if they had missing values for covariates. Therefore, the analysis was performed on the complete dataset (sample sizes: 2007: n=7408; 2009: n=4980; 2011: n=4362; 2013: n=3991; 2016: n=3122). Table 1 presents descriptive statistics of the variables at the first and last waves, as the composition of the analytic sample has not significantly changed over time, except for changes in the distribution of age and the percentage of retired participants. Categorical variables were reported as percentages, while continuous variables were summarised using means and SD. Skewed data were presented as medians with IQRs (25th–75th percentile). At baseline, the mean age of participants was 51 years, which increased to 61 years by the fifth wave. The percentage of retired participants increased from 8.9% to 33.1%.

**Table 1:** Percentages for categorical variables and means (with SD) or medians (with 25th–75th percentiles) for continuous variables at wave 1 and wave 5

Variable	Wave 1	Wave 5
	Total	Total
	n=7408	n=3122
Age	51.1 (7.03)	61.1 (6.97)
Sex		
Male	44.2	41.9
Female	55.8	58.1
Education		
Bachelor’s degree or higher	31.5	38.1
Diploma/associate diploma	11.3	11.9
Vocational (trade/business)	17.6	16.8
School	39.6	33.3
Occupation		
Managers and professionals	33.2	30.2
White collar	22.2	15.9
Blue collar	14.7	8.39
Home duties	6.09	4.23
Retired	8.94	33.1
Missing	14.9	8.2
Annual income		
$A130 000 or more	16.6	19.7
$A72 800–$A129 999	26.1	23.9
$A52 000–$A72 799	14.6	12.6
$A26 000–$A51 999	19.1	18.3
$A0–$A25 999	9.44	11.1
Missing	14.2	14.3
Length of residence at current address (years)*	9.5 (4–18)	11.5 (6–20)
Neighbourhood preference	54.8 (18.4)	54.8 (18.1)
Neighbourhood disadvantage	57.1 (28.1)	57.6 (27.6)
Residential density*	15.2 (13.1–17.7)	15.7 (13.8–19.9)
Street connectivity*	9 (2–18)	12 (6–21)
Land-use mix	0.4 (0.1)	0.4 (0.1)
Transport walkers	34.8	42.4

* Median (25th–75th percentile) is presented instead of mean (SD) as they are positively skewed.

The dataset has a multilevel structure, with repeated observations over time (level 1) nested within participants (level 2), and participants nested within neighbourhoods (level 3). Accordingly, we fitted three-level generalised linear mixed-effects models with a logit link for the binary outcome of transport walking. The models included fixed effects for residential density, street connectivity, land-use mix and walkability, as well as for individual- and neighbourhood-level covariates (age, sex, education, occupation, household income, length of residence, neighbourhood preference and neighbourhood disadvantage). Age, sex, education, length of residence and neighbourhood preference were assessed at baseline, while other covariates and exposures varied over time. The models included random intercepts at both the individual and neighbourhood levels to account for within-participant correlation of repeated measures over time and clustering of participants within neighbourhoods. The regression equation for the fully adjusted main-effects model (model 5 in table 2) is provided in online supplemental equation S1. All models were estimated using the ‘glmer()’ function in the ‘lme4’ package in R software.

**Table 2:** Generalised linear mixed-effects logistic regression models showing the main effects of residential density, street connectivity, land-use mix and walkability on transport walking (walker vs non-walker)

Variable	Model 1	Model 2	Model 3	Model 4	Model 5
	Residential density	Street connectivity	Land-use mix	Walkability	Full model
	OR (95% CI)	OR (95% CI)	OR (95% CI)	OR (95% CI)	OR (95% CI)
Intercept	0.52 (0.31 to 0.88)*	0.71 (0.42 to 1.19)	0.48 (0.26 to 0.90)*	0.31 (0.18 to 0.54)*	0.4 (0.22 to 0.73)*
Main effects					
Residential density	1.04 (1.03 to 1.05)*				1.03 (1.02 to 1.04)*
Street connectivity		1.03 (1.02 to 1.03)*			1.02 (1.01 to 1.02)*
Land-use mix			5.66 (2.66 to 12.05)*		2.50 (1.23 to 5.10) *
Walkability				1.04 (1.04 to 1.05)*	
Covariates					
Age at baseline (year 2007)	0.98 (0.97 to 0.99)*	0.98 (0.97 to 0.99)*	0.98 (0.97 to 0.99)*	0.98 (0.97 to 0.99)*	0.98 (0.97 to 0.99)*
Sex (ref: male)					
Female	0.71 (0.64 to 0.80)*	0.75 (0.67 to 0.84)*	0.71 (0.64 to 0.80)*	0.72 (0.64 to 0.8)*	0.71 (0.63 to 0.79)*
Education (ref: bachelor’s degree or higher)					
Diploma/associate diploma	0.68 (0.57 to 0.82)*	0.68 (0.57 to 0.82)*	0.65 (0.54 to 0.79)*	0.65 (0.54 to 0.79)*	0.68 (0.57 to 0.82)*
Vocational (trade/business)	0.51 (0.43 to 0.60)*	0.47 (0.40 to 0.56)*	0.46 (0.39 to 0.55)*	0.47 (0.40 to 0.56)*	0.50 (0.42 to 0.59)*
School	0.42 (0.37 to 0.49)*	0.41 (0.35 to 0.47)*	0.41 (0.35 to 0.47)*	0.41 (0.35 to 0.47)*	0.44 (0.38 to 0.51)*
Occupation (ref: managers and professionals)					
White collar	1.18 (1.03 to 1.35)*	1.15 (1.00 to 1.31)*	1.16 (1.02 to 1.33)*	1.20 (1.05 to 1.38)*	1.13 (0.99 to 1.29)
Blue collar	0.67 (0.57 to 0.79)*	0.66 (0.56 to 0.79)*	0.68 (0.57 to 0.80)*	0.69 (0.58 to 0.81)*	0.64 (0.54 to 0.76)*
Home duties	0.85 (0.69 to 1.05)	0.87 (0.70 to 1.07)	0.85 (0.69 to 1.05)	0.89 (0.72 to 1.10)	0.82 (0.66 to 1.02)
Retired	0.99 (0.85 to 1.15)	0.98 (0.84 to 1.13)	1.02 (0.87 to 1.18)	1.05 (0.91 to 1.22)	0.98 (0.84 to 1.14)
Other/missing	1.13 (0.98 to 1.30)	1.11 (0.97 to 1.28)	1.13 (0.98 to 1.30)	1.17 (1.01 to 1.34)*	1.13 (0.98 to 1.30)
Income (ref: $130 000 or more)					
$72 800–$129 999	1.09 (0.96 to 1.23)	1.06 (0.93 to 1.20)	1.12 (0.98 to 1.27)	1.11 (0.97 to 1.26)	1.08 (0.95 to 1.22)
$52 000–$72 799	1.00 (0.85 to 1.16)	0.97 (0.84 to 1.14)	1.02 (0.87 to 1.19)	1.02 (0.88 to 1.19)	0.99 (0.85 to 1.16)
$26 000–$51 999	0.92 (0.79 to 1.08)	0.87 (0.74 to 1.01)	0.93 (0.80 to 1.09)	0.93 (0.79 to 1.08)	0.90 (0.77 to 1.05)
$0–$25 999	0.98 (0.82 to 1.19)	0.94 (0.78 to 1.13)	0.99 (0.82 to 1.20)	1.02 (0.84 to 1.22)	0.98 (0.81 to 1.18)
Missing	0.89 (0.76 to 1.04)	0.83 (0.71 to 0.97)*	0.89 (0.76 to 1.04)	0.88 (0.76 to 1.03)	0.86 (0.74 to 1.01)
Neighbourhood disadvantage	1.00 (1.00 to 1.00)	1.00 (1.00 to 1.00)	1.00 (1.00 to 1.00)	1.00 (1.00 to 1.00)	1.00 (1.00 to 1.00)
Neighbourhood preference	1.03 (1.02 to 1.03)*	1.03 (1.02 to 1.03)*	1.03 (1.02 to 1.03)*	1.03 (1.02 to 1.03)*	1.02 (1.02 to 1.03)*
Length of stay	1.00 (1.00 to 1.01)	1.00 (1.00 to 1.01)	1.00 (1.00 to 1.01)	1.00 (1.00 to 1.01)	1.01 (1.00 to 1.01)

* Significant at 5% level of significance.

We tested the main effects of residential density, street connectivity, land-use mix and walkability on transport walking, as well as their interaction effects with age. Given that interaction effects can be complex to interpret, we visualised these relationships using interaction plots for three different age groups (mean age, and 1 SD above and below the mean age) to identify potential interactions.

Model fit was assessed with simulation-based residual diagnostics using ‘*DHARMa*’ package in R.^37^ The dataset is unbalanced, with participants contributing differing numbers of observations across waves. The mixed-effects modelling approach accommodates this unbalanced structure while accounting for within-participant correlation of time-varying observations, and yields valid likelihood-based inference, conditional on observed covariates and random effects.^38^

## Results

Table 2 presents regression results of the main effects of residential density, street connectivity, land-use mix and walkability on transport walking. All these neighbourhood attributes were positively associated with the likelihood of being a transport walker. Specifically, greater levels of residential density (model 1: OR 1.04; 95% CI 1.03 to 1.05), street connectivity (model 2: OR 1.03; 95% CI 1.02 to 1.03), land-use mix (model 3: OR 5.66; 95% CI 2.66 to 12.05) and walkability (model 4: OR 1.04; 95% CI 1.04 to 1.05) were associated with higher odds of being a transport walker. When all neighbourhood attributes were included in the model (model 5), the effect size of land-use mix was substantially attenuated compared with the other two attributes, density and connectivity; however, all three attributes remained statistically significant.

Table 2 also presents results for associations between covariates and transport walking. Older age was associated with a lower likelihood of transport walking, and females had lower odds of being transport walkers compared with males. Lower levels of education were associated with reduced odds of transport walking. Similarly, individuals in blue-collar occupations had lower odds of transport walking compared with those in managerial and professional roles, while differences for other occupational groups were less consistent across models. Household income, neighbourhood disadvantage and length of residence showed no significant associations with transport walking. Although the effect was modest, neighbourhood preference was positively and significantly associated with transport walking across all models, indicating that individuals who preferred walkable environments were more likely to engage in transport walking. Adjusting for this measure helps reduce confounding due to residential preference and yields more conservative estimates of the associations between built environment attributes and transport walking.

**Figure 1:**
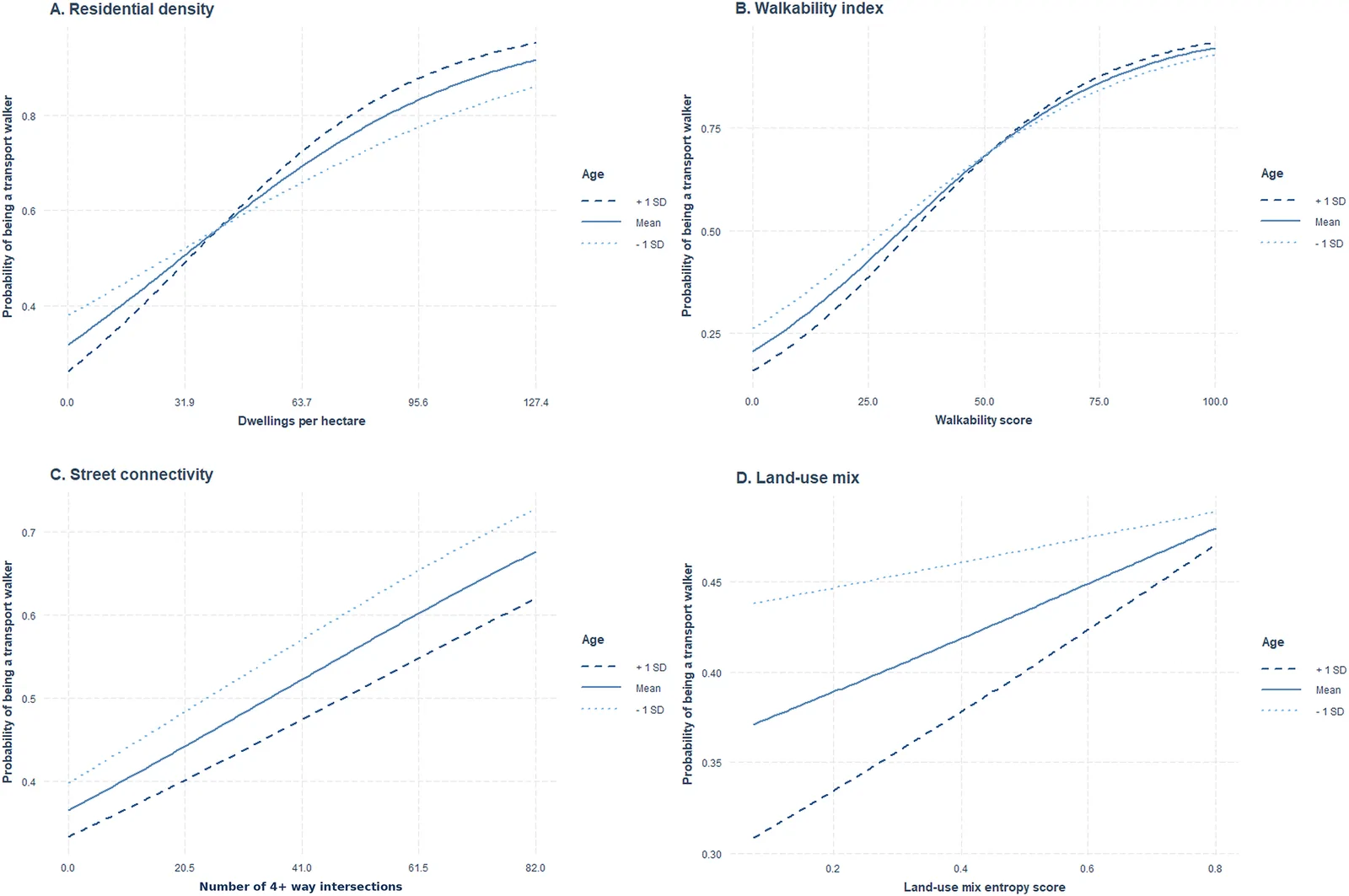
Predicted likelihood of being a transport walker by residential density (A), walkability (B), street connectivity (C) and land-use mix (D) across three different age groups (mean age and 1 SD above and below the mean age).

Only residential density (OR 1.001; 95% CI 1.000 to 1.002) and walkability (OR 1.001; 95% CI 1.000 to 1.002) showed significant interaction effects with age on transport walking. This is clearly indicated in figure 1, showing that older adults were more likely to be transport walkers in neighbourhoods with high residential density (plot A) or high walkability (plot B).

## Discussion

This study explored the longitudinal effects of residential density, street connectivity, land-use mix and walkability on transport walking, and their interaction effects with age. We found that higher levels of these built environment attributes were associated with a greater likelihood of any transport walking among middle- to older-aged residents of Brisbane, Australia. These findings strengthen the positive associations found in the literature. A recent systematic review of longitudinal studies found that street connectivity is a strong determinant of transport walking.^16^ A cross-sectional analysis by Cerin *et al*^39^ found that high-density environments support within-neighbourhood transport walking. The authors also revealed that extreme density can lead to a reduction in walking. The non-linear form of this association is also evident in our study (see plot A in figure 1).

Only residential density and walkability showed significant interaction effects with age on transport walking. Several factors may explain why older adults appear to benefit more from high-density, walkable neighbourhoods. Higher residential density is often associated with greater availability of public spaces and local services, which can facilitate social interaction and community engagement and support psychological well-being.^40,41^ Opportunities for spontaneous social encounters may motivate older adults to leave home and walk locally, helping to reduce loneliness and enhance quality of life.^42^ In addition, high walkability—characterised by proximity to shops, services and public transport—may help mitigate age-related mobility limitations by enabling older adults to meet daily needs within shorter walking distances. Non-significance of the interaction effect of street connectivity and age on transport walking was supported by the parallel lines in the interaction plot (see plot C in figure 1). Although the interaction effect between land-use mix and age was not statistically significant, the interaction plot revealed a large difference in the probability of being a transport walker between younger and older adults at low levels of land-use mix compared with high levels (see plot D in figure 1).

This study has several key strengths. The study strengthens temporal inference on the associations explored due to its longitudinal design, with five waves of data collected over 9 years. By incorporating time-varying environmental measures and confounders that were collected alongside transport walking data, the study enhances the reliability of the measurements and temporality of the associations.^19^ The inclusion of a proxy measure for participants’ preferences for living in walkable neighbourhoods helped to address potential residential self-selection bias in the association between neighbourhood environment and transport walking. Additionally, the use of objectively measured neighbourhood attributes strengthens the study’s methodological rigour and reduces potential biases associated with self-reported data, enhancing the reliability of the findings.

Despite its strengths, this study has several limitations. First, transport walking was self-reported and subject to recall bias^43^; however, the binary classification of walkers versus non-walkers may have reduced the impact of any recall bias relative to recalling walking duration. Second, although dichotomisation facilitated robust modelling of highly skewed and zero-inflated data as is commonly done in research in this field,^44^ it does not capture variation in the amount of walking. Alternative analytical approaches—such as modelling continuous or ordinal measures of walking duration—are also appropriate and may provide additional insights. The present study adopted a dichotomous outcome as a pragmatic approach that is widely used in the literature and allows for straightforward interpretation and comparison with prior studies.^10,45,46^ Future research could extend this work by applying alternative modelling strategies to better capture variation in the amount of transport walking. Third, the analytic sample was restricted to participants who remained at the same residential address to reduce potential bias arising from residential mobility, as residential relocation can be influenced by a wide range of factors beyond the scope of this study, including health and housing conditions.^47,48^ While this approach strengthens internal validity, it may limit generalisability to individuals who relocate. Finally, although sample attrition across waves has resulted in a slight overrepresentation of participants from higher socioeconomic groups,^20^ the HABITAT cohort was broadly representative of the Brisbane population aged 40–65 years at baseline and remained broadly similar in profile at later waves.^20,21^ While attrition was socially patterned, key socioeconomic indicators were adjusted for in all models, including education, occupation, income and neighbourhood disadvantage. Therefore, the findings can be generalised to the Brisbane population aged 40–65 years, including disadvantaged groups. Nevertheless, caution is warranted when generalising findings to socioeconomically disadvantaged populations in other countries.

Our findings have important implications for urban planning and public health policies aimed at promoting transport walking, particularly among middle-aged and older adults, to increase physical activity and support healthy ageing. The positive associations between walkability-related attributes and transport walking highlight the need for city designs that prioritise well-connected streets, mixed land-use developments and higher residential densities. Moreover, the observed age differences in the associations of residential density and walkability with transport walking suggest that policies seeking to promote walking among older adults should place particular emphasis on creating dense, walkable neighbourhoods. Although the cohort was aged 40–65 years at baseline (mean age 51 years), participants aged into later life over follow-up (mean age 61 years). Nevertheless, the findings may not fully generalise to adults aged 65 years and older, who often face more pronounced mobility limitations that influence walking behaviour.^49^ For this older population, additional built environment and social characteristics - such as benches, presence of pedestrian crossings, and safety from crime—may be particularly important for supporting transport walking.^45,50^ Future research focusing specifically on adults aged 65 years and over is needed to examine whether the benefits of density and walkability persist or whether other environmental attributes become increasingly important with advancing age.

## Conclusion

This study provides longitudinal support for the influence of built environment attributes—residential density, street connectivity, land-use mix and walkability—on transport walking among mid- to older-aged adults in Brisbane, Australia. The findings highlight the importance of promoting higher residential density and overall walkability to support transport walking, particularly for older adults. This study’s longitudinal design, use of time-varying built environment measures across 9 years, and adjustment for residential self-selection bias add to the strength of the evidence. Future research should address the limitations of self-reported transport walking data and further investigate the dynamic, non-linear relationships between built environment attributes and transport walking behaviours, particularly across different age cohorts. Policymakers can leverage these findings to design walkable neighbourhoods that encourage active lifestyles and promote public health across the lifespan.

## Supplementary material

10.1136/bmjph-2025-003333 online supplemental file 1

## Data Availability

No data are available.
