# Supplementary material for: What neighbourhood factors support more transport walking as adults age? A five-wave longitudinal multilevel cohort study in Brisbane, Australia
Source: BMJ Public Health. 2026 Sep 4;4(3):e003333. doi: 10.1136/bmjph-2025-003333 (PMC13548265; doi:10.1136/bmjph-2025-003333)
Supplement: Supplementary file 1 [file bmjph-4-3-s001.docx]

# Supplementary file

**Equation S1**: Regression model structure of the main effects full model (Model 5 in Table 2).

$$Logit\left[ \Pr\left( Y_{ijt}=1 \right) \right]=\beta_{0}+ \beta_{1}Age_{i}+ \beta_{2}Sex_{i}+ \beta_{3}Edu_{i}+ \beta_{4}NeighPref_{i}+ \beta_{5}LenRes_{i}+ \beta_{6}Occu_{it}+ \beta_{7}Income_{it}+ \beta_{8}ResDen_{it}+ \beta_{9}StreetCon_{it}+ \beta_{10}LandUse_{it}+ \beta_{11}NeighDis_{jt}+ u_{i}+ v_{j}$$

$$Y_{ijt}= \left\{ \begin{aligned} 1: walked \\ 0:did not walk \end{aligned} \right.$$

$\beta_{0}$ is the fixed intercept

$\beta_{1}\ldots\beta_{11}$ are fixed-effect coefficients

$u_{i}\sim N(0,\sigma_{u}^{2})$ is the individual-level random intercept

$v_{j}\sim N(0,\sigma_{v}^{2})$ is the neighbourhood-level random intercept

| **Variable** | **Description** |
| --- | --- |
| $Age_{i}$ | Baseline age of participant i. |
| $Sex_{i}$ | Sex of participant i. |
| $Edu_{i}$ | Education level of participant i. |
| $NeighPref_{i}$ | Neighbourhood preference score (neighbourhood self-selection proxy measure) of participant i. |
| $LenRes_{i}$ | Length of residence of participant i in years. |
| $Occu_{it}$ | Occupation category of participant i at wave t. |
| $Income_{it}$ | Income category of participant i at wave t. |
| $ResDen_{it}$ | Residential density (within 1km network buffer) of participant i at wave t. |
| $StreetCon_{it}$ | Street connectivity (within 1km network buffer) of participant i at wave t. |
| $LandUse_{it}$ | Land use mix (within 1km network buffer) of participant i at wave t. |
| $NeighDis_{jt}$ | Neighbourhood disadvantage score of neighbourhood j at wave t. |

**Table S1**: HABITAT study survey questions.

| Measure of this study | HABITAT survey question |
| --- | --- |
| Transport walking |  |
| Age |  |
| Sex |  |
| Education |  |
| Occupation |    |
| Household income |  |
| Length of residence |  |
| Neighbourhood preference |  |
